# Targeting cancer-derived extracellular vesicles by combining CD147 inhibition with tissue factor pathway inhibitor for the management of urothelial cancer cells

**DOI:** 10.1186/s12964-024-01508-x

**Published:** 2024-02-15

**Authors:** Vijay Kumar Boddu, Piet Zamzow, Mario Wolfgang Kramer, Axel S. Merseburger, Sivahari Prasad Gorantla, Matthias Klinger, Lena Cramer, Thorben Sauer, Timo Gemoll, Nikolas von Bubnoff, Frank Gieseler, Masoud Darabi

**Affiliations:** 1 Department of Hematology and Oncology, Section for Experimental Oncology, University Hospital Schleswig-Holstein, Lübeck, Germany; 2 Department of Urology, University Hospital Schleswig-Holstein, Lübeck, Germany; 3 Department of Hematology and Oncology, University Hospital Schleswig-Holstein, Lübeck, Germany; 4 Institute of Anatomy, University of Lübeck, Lübeck, Germany; 5 Department of Surgery, Section for Translational Surgical Oncology and Biobanking, University Hospital Schleswig-Holstein, Lübeck, Germany; 6 University Cancer Center Schleswig-Holstein (UCCSH), Lübeck, Germany

**Keywords:** Bladder carcinoma, CD142, EMMPRIN, Microvesicles

## Abstract

**Background:**

Extracellular vesicles (EVs), including microvesicles, hold promise for the management of bladder urothelial carcinoma (BLCA), particularly because of their utility in identifying therapeutic targets and their diagnostic potential using easily accessible urine samples. Among the transmembrane glycoproteins highly enriched in cancer-derived EVs, tissue factor (TF) and CD147 have been implicated in promoting tumor progression. In this in vitro study, we explored a novel approach to impede cancer cell migration and metastasis by simultaneously targeting these molecules on urothelial cancer-derived EVs.

**Methods:**

Cell culture supernatants from invasive and non-invasive bladder cancer cell lines and urine samples from patients with BLCA were collected. Large, microvesicle-like EVs were isolated using sequential centrifugation and characterized by electron microscopy, nanoparticle tracking analysis, and flow cytometry. The impact of urinary or cell supernatant-derived EVs on cellular phenotypes was evaluated using cell-based assays following combined treatment with a specific CD147 inhibitor alone or in combination with a tissue factor pathway inhibitor (TFPI), an endogenous anticoagulant protein that can be released by low-molecular-weight heparins.

**Results:**

We observed that EVs obtained from the urine samples of patients with muscle-invasive BLCA and from the aggressive bladder cancer cell line J82 exhibited higher TF activity and CD147 expression levels than did their non-invasive counterparts. The shedding of GFP-tagged CD147 into isolated vesicles demonstrated that the vesicles originated from plasma cell membranes. EVs originating from invasive cancer cells were found to trigger migration, secretion of matrix metalloproteinases (MMPs), and invasion. The same induction of MMP activity was replicated using EVs obtained from urine samples of patients with invasive BLCA. EVs derived from cancer cell clones overexpressing TF and CD147 were produced in higher quantities and exhibited a higher invasive potential than those from control cancer cells. TFPI interfered with the effect when used in conjunction with the CD147 inhibitor, further suppressing homotypic EV-induced migration, MMP production, and invasion.

**Conclusions:**

Our findings suggest that combining a CD147 inhibitor with low molecular weight heparins to induce TFPI release may be a promising therapeutic approach for urothelial cancer management. This combination can potentially suppress the tumor-promoting actions of cancer-derived microvesicle-like EVs, including collective matrix invasion.

**Graphical Abstract:**

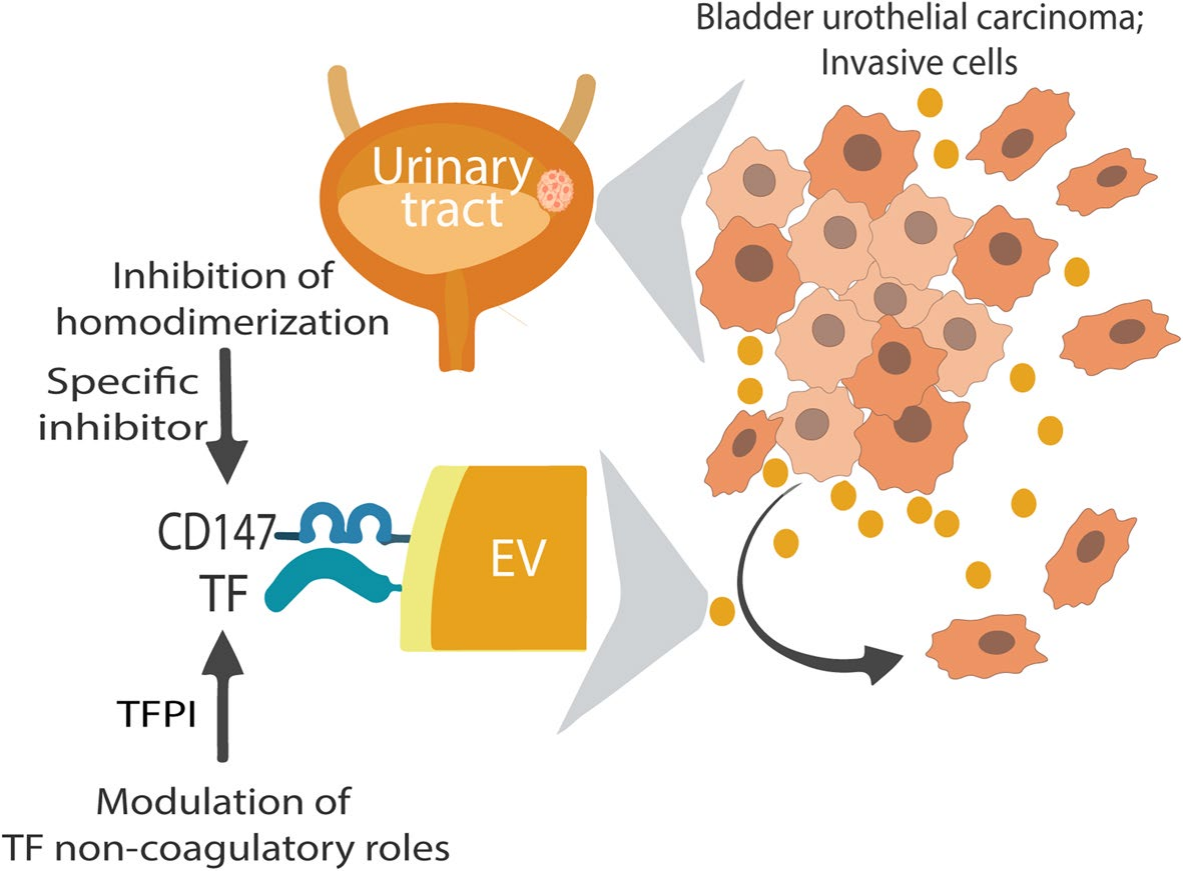

**Supplementary Information:**

The online version contains supplementary material available at 10.1186/s12964-024-01508-x.

## Highlights

• Invasive bladder cancer cells shed extracellular vesicles (EVs) that contain tissue factor (TF) and CD147.

• Tumor-derived EVs can promote cancer cell migration and invasion.

• A combination of a CD147 inhibitor and tissue factor pathway inhibitor (TFPI) can suppress the cancer-promoting actions of EVs.

**Supplementary Information:**

The online version contains supplementary material available at 10.1186/s12964-024-01508-x.

## Plain summary

Small particles or vesicles released by cancer cells into their surroundings have the potential to stimulate the spread and growth of cancer cells. In this study, we focused on two specific molecules presented by these cancer cell-derived vesicles that could play a role in promoting the dissemination of cancer cells: a protein related to blood clotting and a protein on the cell surface.

We found that large vesicles from bladder cancer cells that have the ability to spread had higher levels of these proteins than vesicles from nonspreading cancer cells. We also found that the former could make cancer cells move about more, produce more of a substance that helps cancer cells spread, and invade other tissues.

To counteract the cancer-promoting actions of these vesicles, we examined the impact of combining a naturally occurring anticlotting protein that can be released by medications derived from heparin with an inhibitor targeting the cancer cell surface protein. We found that this combination stopped the vesicles from helping cancer cells move about more, produce more of the spreading substance, and invade other tissues.

This approach of simultaneously targeting the two protein molecules present on cancer cell-derived vesicles might be a new way to treat bladder cancer.

**Supplementary Information:**

The online version contains supplementary material available at 10.1186/s12964-024-01508-x.

## Background

Cancer cells employ various extracellular communication mechanisms to drive their progression and metastasis [1]. While the plasma membrane has been recognized as the primary interface for interactions with the extracellular environment, cancer cells also utilize a type of extracellular vesicles (EVs) called microvesicles or ectosomes for intercellular communication [2]. Unlike exosomes, which originate from endosomal organelles, microvesicles are directly shed from the plasma membrane. They exhibit a broader size range and have the potential for direct interaction with neighboring cells through their membrane [3]. This distinct characteristic allows microvesicles to exert remote signaling effects by dissemination through body fluids [4].

Extensive research has indicated that intercellular communication is partially facilitated by the presence of genetic materials, particularly RNAs, packaged within secretory vesicles (reviewed in [5]. However, it is crucial to note that the impact of genetic materials is often delayed and constrained when compared to that of membrane structures. This is primarily due to the necessity of content transfer and the potential need for further processing within recipient cells before exerting an effect.

Recent evidence suggests that tumor-derived EVs play a crucial role in tumor progression by acting via multiple mechanisms to trigger invasion and metastasis. EVs mediate both homotypic targeting of tumor cells [6, 7] and heterotypic interactions with the tumor microenvironment [8–10].

Tissue factor (TF), also known as Factor III, CD142, or thromboplastin, is a transmembrane glycoprotein with a well-established role in blood coagulation. We have demonstrated that TF-bearing microvesicles can induce tumor cell migration, highlighting their potential role in cancer progression [11]. Additionally, elevated levels of TF associated with EVs have been correlated with tumor aggressiveness and increased mortality among cancer patients [12].

Another transmembrane glycoprotein highly incorporated into cancer EVs is the extracellular matrix metalloproteinase inducer (CD147/EMMPRIN) [13]. Studies have shown that CD147 is upregulated in various cancers and plays a role in tumor invasion and metastasis [14–16].

Due to their unique properties, EVs have emerged as promising candidates for bladder carcinoma management. First, EV-associated TF levels have been shown to be correlated with thrombosis, a common complication in cancer patients, thereby providing valuable information on patient prognosis. Second, EVs can carry molecules that are clinically relevant to tumor cell properties, such as infiltration and metastasis, making them valuable tools for understanding disease progression. Finally, EVs have diagnostic potential because they can be easily isolated from urine samples, which is a non-invasive and cost-effective method for diagnosis and monitoring bladder cancer.

Inhibitors of CD147, a transmembrane glycoprotein, and TFPI, a natural inhibitor of the coagulation pathway, have shown potential implications for inhibiting tumor progression, metastasis, and chemoresistance [17–19]. In a previous study, we showed that TFPI, which is released in response to the low-molecular-weight heparins (LMWHs) Tinzaparin, can suppress cancer cell progression induced by cancer-derived EVs [20]. In this study, we investigated whether the combination of TFPI with a CD147 inhibitor greater effect on EV-induced bladder cancer cell migration and metastasis, than does inhibition of CD147 alone.

## Methods

**Fig. 1:**
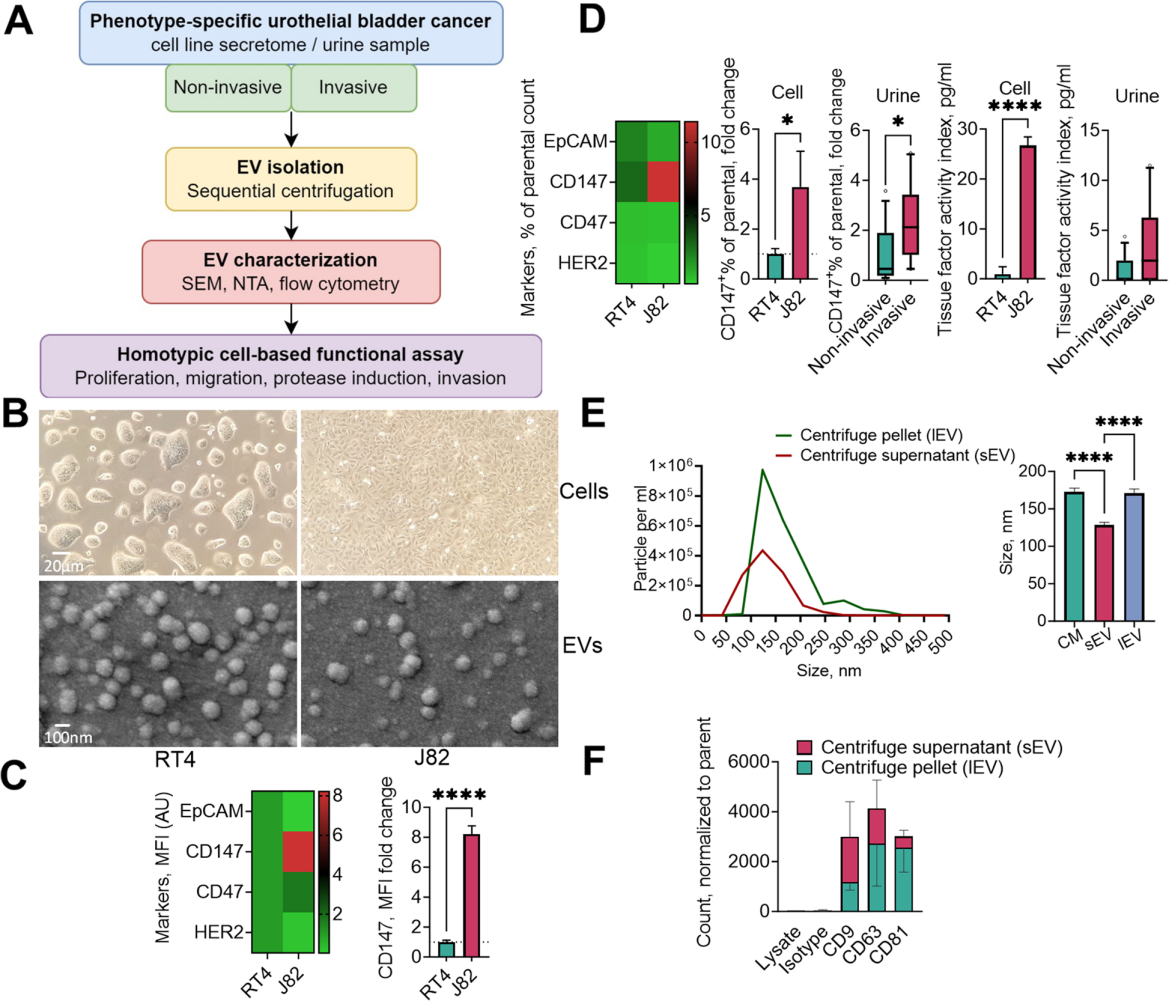
Workflow of sample processing and experimental design. Conditioned media were collected over a 24 h incubation period from two human cell lines, RT4 and J82, grown in monolayer culture. Urine samples were collected from patients with bladder urothelial carcinoma. Extracellular vesicles (EVs) were isolated and characterized using scanning electron microscopy (SEM), nanoparticle tracking analysis (NTA), and high-resolution flow cytometry. The homotypic effects of EVs were evaluated via cell-based functional assays with different readouts of cellular phenotypes (**A**). Cell and EV morphology were visualized using light microscopy and SEM, respectively (**B**). Cells (**C**) and cell- and urine-derived Evs (**D**) were analyzed for surface markers. The particle number and size distribution of the EVs were analyzed in unfractionated conditioned medium, centrifugation supernatant containing small EVs (sEVs), and centrifugation pellet containing large, microvesicle-like EVs (lEVs) by NTA (**E**), and the presence of tetraspanin EV markers was analyzed by flow cytometry (**F**) of both the EV pellet and centrifuge supernatant derived from muscle-invasive J82 cells. The parameters of the urine-derived EVs are presented as medians with 10th and 90th percentiles and were compared using the non-parametric Mann–Whitney U test. MFI, mean fluorescence intensity

This study evaluated the homotypic functional effect of microvesicle-like EVs in cell-based assays (Fig. 1A). EVs were isolated from the supernatant of human adherent bladder cell lines grown in EV-free conditions or from urine samples obtained from patients with bladder urothelial carcinoma (BLCA).

### Cell lines and culture conditions

We utilized two urinary bladder cancer cell lines: RT4 (CLS GmbH, Germany), which is classified as non-muscle invasive (low grade), and J82 (ATCC HTB-1), which is classified as muscle-invasive (high grade). Both cell lines exhibit adherent growth, with doubling times of 40 h for RT4 and 24 h for J82.

The cells were grown in RPMI-1640 medium supplemented with 10% FBS and incubated at 37°C in 5% CO_2_ and 95% humidity to maintain optimal growth conditions. Regular tests for mycoplasma contamination were performed, and the cell lines were confirmed to be free of contamination. Prior to EV collection or treatment, the cultures were washed with PBS, after which the medium was replaced with medium supplemented with 5% exosome-depleted FBS (Gibco, Germany) to maintain cell viability. The exosome-depleted FBS was certified by the manufacturer to be devoid of detectable EV marker CD63, addressing concerns about EV contamination in ultracentrifuged exosome-depleted serum [21]. Moreover, the resulting protein pattern was similar to that of the source FBS.

J82 cells were exposed to their own isolated and characterized microvesicle-like EVs, which were pre-incubated with or without the CD147 inhibitor AC-73 (MedChemExpress LLC, NJ), human recombinant TFPI together with factor VII (Sigma-Aldrich, MO), or human recombinant CD147 protein (rCD147, Sino Biological Europe, Germany) for the specified duration.

### Plasmids and transfection

CD147 constructs [22] were kindly provided by Dr. Sandrine Bourdoulous (Université Paris Descartes, France). The TF plasmid [23] was a gift from Dr. Alexander McLellan through Addgene (Watertown, MA). For protein expression in mammalian cells, cells were transfected using FuGENE HD Transfection Reagent (Promega, WI), according to the manufacturer's protocol.

### Human sample collection

A total of 27 patients diagnosed with urothelial malignancies were enrolled in the study (Supplementary Table 1). A subset of patients had to be excluded due to limitations in sample volume from liquid biopsies. These individuals were referred to our study due to suspected bladder carcinoma and subsequently underwent transurethral resection of the bladder tumor. At the time of their clinic visit, all patients were requested to provide a urine sample. The study protocol received approval from the local ethics committee under code 22–240, and all patients were provided with detailed information by the urology team and signed consent forms.

Following diagnosis, we reviewed the bladder biopsy pathology findings for each patient and documented the malignant findings into subgroups, distinguishing invasive from non-invasive tumors, despite the lack of detailed TNM staging information for each individual.

### Isolation of microvesicle-like EVs by sequential centrifugation

Cell-free 24-h conditioned medium or urine was prepared by centrifuging the samples at 470 × g for 10 min and storing them at -80°C until further use. Conditioned medium was collected from cells at approximately 85% confluence. Microvesicle-like EVs were isolated using a sequential centrifugation method as previously described (Ender, Zamzow, Bubnoff, & Gieseler, 2019). In brief, 1 ml aliquots of conditioned medium or urine were centrifuged twice at 2,500 × g for 15 min at 4°C using a microcentrifuge (Eppendorf AG, Germany). The supernatant was collected, and high-speed centrifugation susequently was carried out at 10,000 × g for 1.5 h at 4°C using the same centrifuge. The pellet was collected and resuspended in 0.22 µm pore-size-membrane (Merck Millipore Ltd., Ireland) filtered PBS.

### Scanning electron microscopy

For scanning electron microscopy (SEM), the EV pellet was resuspended in 4% paraformaldehyde and incubated at room temperature for 20 min. The EV sample was then washed in water and centrifuged at 10,000 × g for 1.5 h, after which the pellet was resuspended in distilled water. SEM was conducted as previously described [24], involving EV sample application, drying, and gold coating. Finally, the EVs were imaged using an EVO LS15 scanning electron microscope (Carl Zeiss AG, Germany).

### Nanoparticle tracking analysis

All the microparticle analyses were performed with a NanoSight NS300 (Malvern Instruments Ltd., UK) instrument equipped with a 488 nm laser module. All the samples were diluted in PBS and filtered through 0.22 µm membrane filters (Millex, Merck Millipore Ltd., Ireland) to a final volume of 1 ml. All the measurements were taken in replicates of at least 3 for each sample, simultaneously capturing 30-s videos with a minimum number of valid tracks recommended according to the manual. NanoSight NTA software was used for all the analyses. The camera level for each sample was manually adjusted to attain the ideal visualization of particles, and the sample infusion rate was set at 100 µL/minute. The particle size was assessed regularly using 100 nm polystyrene beads (Malvern).

### Confocal microscopy

J82 cells were fixed with 4% paraformaldehyde 24 h after transfection. Subsequently, the cells on coverslips were mounted using a medium containing the nuclear dye Hoechst 33,342 (Thermo Scientific, MA) for nuclei counterstaining. The cells were imaged using a confocal microscope (Olympus FV1000, Japan) with an oil 60 × objective.

### Flow cytometry

We utilized a high-resolution Novocyte flow cytometer (ACEA Biosciences Inc., CA) to characterize the size and concentration of microvesicle-like larger EVs isolated by high-speed centrifugation. To confirm the integrity of the EVs, we used the membrane-permeable fluorescent dye carboxyfluorescein diacetate succinimidyl ester (CFDA-SE; Cayman Chemical, MI), as described previously [25]. The samples were incubated with 40 µM CFSE for 10 min at room temperature in the dark. The samples were then incubated with antibodies conjugated with phycoerythrin (PE) and detected via the FITC and PE channels for both CFSE and antibody staining, respectively. We employed size gates ranging from 0.1 to 0.9 µm, a method previously described by our laboratory [26], using MegaMix Beads (BioCytex, France) to determine the size of the extracellular vesicle (EV) population.

The expression of surface markers with potential clinical relevance in bladder cancer, namely, EpCAM, CD147, CD47, and HER2, was determined on both cells and isolated EVs. For the staining procedure, we collected an equal number of cells or EVs from both conditioned medium and bladder cancer samples and immunostained them with labeled antibodies. Fluorescence minus one (FM-O) controls of the same samples without staining of interest were used to assess the expression levels. The samples were incubated with antibodies for 20 min at 4°C following CFSE labeling and then washed with 1 ml of PBS.

### Tissue factor activity assay

A Zymuphen MP-TF assay kit (Aniara, OH) was used to measure TF activity as previously described [24]. The assay was based on the principle of TF-mediated conversion of the substrate to its active form. In a 96-well microplate, the diluted sample or standard was pipetted in duplicate. Subsequently, the substrate solution from the kit was added to each well, and the microplate was sealed and incubated at room temperature overnight. The next day, the wells were washed, and human factor VIIa and X were added to each well. The plate was sealed and incubated for 2 h at 37°C. Subsequently, chromogenic substrate for factor Xa was added to each well and incubated for 2 h at 37°C. The reaction was stopped by adding stop solution to each well, followed by gentle mixing. The absorbance was measured at 405 nm using an Infinite F200 microplate reader (Tecan, Switzerland). TF activity in the sample was calculated using a standard curve generated from the absorbance values of the standards provided in the kit.

### Cell viability assay

Cell viability was assessed using the MTS tetrazolium reduction assay (CellTiter 96; Promega, WI). Cells were seeded in triplicate into 96-well plates and incubated for the designated times. Subsequently, 10% tetrazolium solution was added to each well, and the plates were further incubated at 37°C for 3 h. During this incubation, viable cells converted the MTS tetrazolium salt into a formazan product. The absorbance of the formazan product was measured using a microplate reader, and the results were recorded as optical density (OD) values, which are directly proportional to cell viability. To calculate the percentage of cell viability, the OD values of the experimental groups were normalized to those of the control group.

### Metalloproteinase activity

Metalloproteinase activity was measured using an AmpliteTM Universal Fluorimetric MMP Activity Assay Kit following the manufacturer's instructions (AAT Bioquest, CA). Briefly, attached cells were stimulated with or without inhibitors, and 24 h conditioned medium was collected to test enzymatic activity in the form of substrate cleavage. To exclude any direct EV-derived MMP activity, a no-cell control was included in the assay. Signals were assessed 2 h after initiating the enzymatic reaction using a microplate reader equipped with a filter set of excitation (Ex)/emission (Em) = 485 nm/535 nm. The amount of substrate conversion, reflecting the MMP activity, was quantified using the relative fluorescence units (RFU) obtained from the assay.

### Proteomics

The global label-free proteome was analyzed by liquid chromatography-coupled mass spectrometry (LC–MS). Protein samples (100 µg each) were prepared using the EasyPep kit (Thermo Fisher, MA). The proteins were processed into dried pellets and reconstituted in 5% formic acid for mass spectrometric analysis. The extracted peptides were loaded onto C18 EvoTip disposable trap columns. Chromatographic separation of 200 ng per sample was performed on an EvoSep One (EvoSep, Denmark) using a C18 Performance column (EV1137, 15 cm × 150 µm, 1.5 µm) in combination with the extended 15 SPD method (88 min gradient, 220 nl/min) [27–29]. For details of the LC–MS acquisition, see the Supplementary methods.

A unique protein matrix was generated using the DIA-NN R package, which included only proteins that were identified and quantified by proteotypic peptides and that passed the FDR cutoff of 0.01. Protein abundance was calculated using the MaxLFQ algorithm [30]. Quantitative data were preprocessed using the R package DEP [31]. The unique protein dataset was filtered for completeness: each protein had to be identified in all replicates of at least one condition/group. Variance stabilizing normalization was applied to the data [32], including log_2_ transformation, before imputing the remaining missing values using a k-nearest neighbor model. Differential expression was evaluated using the limma R package [33]. A net log_2_-fold change  > 1.0 and a Benjamini–Hochberg procedure-adjusted *p*-value < 0.05 were considered to indicate significant differential expression.

### Migration and invasion assays

For the migration and invasion assays, hanging cell culture inserts with a pore size of 8.0 µm polyethylene terephthalate (Millipore, MA) were used in 24-well plates. The inserts were washed twice with medium to prepare the chambers for the assays. For the invasion assay, diluted Matrigel (Corning, VA) was added to the center of each cell well insert and gently spread across the entire membrane surface. The coated inserts were then placed in an incubator at 37°C for 30 min to allow the Matrigel to solidify.

Next, the cell suspensions in serum-free medium, along with EVs or inhibitors, were plated into the upper chambers of both the migration and invasion inserts. Medium supplemented with 10% FBS was added to the lower chamber as a chemoattractant. The cells were then cultured for 16 or 24 h to allow for migration or invasion, respectively.

After the culture period, the medium was aspirated from the lower chambers, and any nonmigrated cells on the upper side of the membrane were carefully removed using a cotton swab. To visualize the migrated and invaded cells, Diff-Quik staining was performed for the inserts using a commercial kit (RAL Diagnostics, France). Briefly, the cells were fixed, rinsed with PBS, and stained with eosin for 1 min. The cells were then counterstained with methylene blue for 1 min. After rinsing off the excess stain, the filters were air-dried. The stained cells were visualized and imaged under a light microscope for analysis. Visual fields on the lower side of the filter were imaged for further analysis. Cell migration and invasion were quantified using ImageJ analysis software. Grayscale images were transformed into binary images through thresholding. Overlapping cells were isolated using Watershed segmentation. The number of nuclei per field was measured using Analyze Particles.

### Extracellular vesicle binding assay

The J82 cell suspension was exposed to different treatment conditions in the presence of CFSE-labeled EVs for 20 min. Flow cytometry was utilized to evaluate the FITC signal across the cell population. The cells were categorized into positive and negative sub-populations based on the attained signal.

### Data analysis

At least three independent experiments were performed for each analysis. Unless otherwise specified, the data are presented as the fold change relative to the control condition and are displayed as mean ± standard deviation (SD). GraphPad Prism was used to create graphs. Mean differences were tested using a t-test or one-way ANOVA. A *p*-value of less than 0.05 was considered to indicate statistical significance. Different significance levels are indicated by 1–3 asterisks corresponding to *p*-values of < 0.05, < 0.01, and < 0.001.

## Results

### Invasive bladder cancer exhibits distinct markers in both cell-derived and urinary large extracellular vesicles

Figure 1B displays the microscopic morphology of the cell lines and their corresponding microvesicle-like EVs. We found that CD147 expression was significantly higher in invasive J82 cells than in non-invasive RT4 cells (Fig. 1C and D). Additionally, we found that, in contrast to those isolated from the non-invasive cohort, EVs isolated from the urine samples of patients with invasive BCLA and J82 cells exhibited significantly elevated TF activity and CD147 expression (Fig. 1D).

Regarding the size distribution of the isolated particles, our observations revealed an average size of 171 nm, with a more restricted distribution range observed for the small EVs remaining in the conditioned medium after centrifugation (Fig. 1E). In both small and large isolated EVs, we detected the presence of the EV markers CD9, CD63, and CD81, with relatively higher enrichment of these markers in large, microvesicle-like EVs (Fig. 1F).

We found that CD147 expression was significantly higher in muscle-invasive J82 cells than in non-invasive RT4 cells (Fig. 1C). Additionally, we found that EVs isolated from urine samples of patients with muscle-invasive BLCA and J82 cells exhibited significantly elevated TF activity and CD147 expression, in contrast to those isolated from the non-invasive set (Fig. 1D).

Regarding the size distribution of the isolated particles, we noted an average size of 120 nm for the J82 cell-derived EVs, which was significantly larger than that of the particles found in the centrifuge supernatant and had a broader distribution range (Fig. 1E). A similar disparity, but in a higher range, was noted for EV subsets derived from RT cancer cells (Supplementary Fig. 2A). We also observed a trend toward enrichment of the tetraspanin proteins CD9, CD63, and CD81 in the isolated larger, microvesicle-like EVs (Fig. 1F and Supplementary Fig. 1B). This further confirmed the identity of the isolated particles used in subsequent functional tests.

### Tagged CD147 sheds into isolated vesicles

**Fig. 2:**
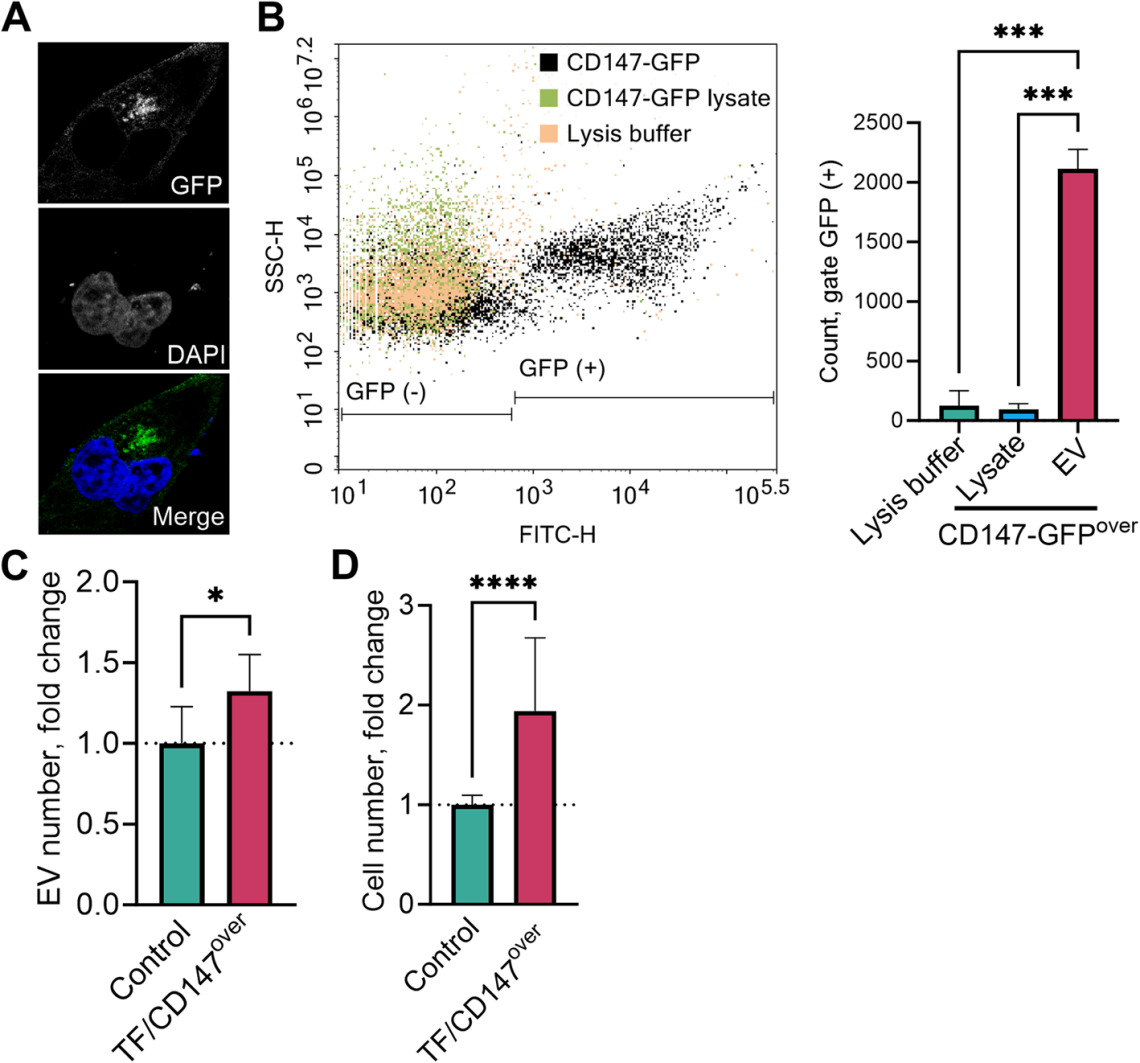
Association of tissue factor (TF) and CD147 overexpression with extracellular vesicle (EV) production and invasion potential. The expression of green fluorescent protein (GFP)-fusion CD147 on the plasma membrane of J82 cells was observed using confocal microscopy (**A**). To assess the vesicular export of CD147, GFP-labeled vesicles were derived from J82 cells transfected with the CD147-GFP construct and analyzed using flow cytometry (**B**). The lysates of the EVs were prepared using 0.1% Triton™ X-100. EVs were derived from human bladder urothelial carcinoma J82 cells co-transfected with TF and CD147 plasmids. The EVs were quantified using NTA in the 48-h culture supernatant of J82 cells overexpressing CD147 and TF versus that of the control transfection condition (**C**). Invasion potency was measured by a Transwell assay, with 6–8 images of the cells that had passed through the coated microporous membrane after 24 h per experiment (**D**)

Microscopic analysis of CD147-GFP expression revealed that CD147 was expressed on the cell surface (Fig. 2A). Moreover, Fig. 2B demonstrates the shedding of GFP-tagged CD147 into isolated vesicles, as indicated by the loss of the GFP signal after detergent treatment. These findings provide additional confirmation of the presence of vesicles derived from the plasma membrane of J82 cells.

### Model EVs with higher levels of TF and CD147 showed enhanced invasion potential

We investigated whether ectopic high expression of TF and CD147 was associated with the level of EV production. Cells overexpressing TF and CD147 produced 32% more EVs than the control mock-transfected cells (*p* = 0.01, Fig. 2C).

Based on the notable increase in TF and CD147 levels in EVs derived from invasive cancer cells and urine samples of patients with invasive BCLA, we investigated the potential link between TF and CD147 overexpression and their functional characteristics using model EVs. Our findings demonstrated that EVs from J82 cancer cell clones overexpressing TF and CD147 had a significantly higher invasive potential compared to EVs from control cancer cells (Fig. 2D).

### CD147 inhibitor and TFPI demonstrate minimal impact on cellular proliferation at conventional levels

Treatment with the CD147 inhibitor AC-73 for 24 h resulted in a significant decrease in J82 cell viability at a high concentration of 20 µM, with only 44% of the cells remaining viable (*p* = 0.002; Supplementary Fig. 2A). However, lower doses of AC-73 with or without TFPI did not produce a statistically significant difference in cell viability compared to untreated control cells. The addition of EVs or inhibitors did not lead to a statistically significant alteration in cellular proliferation, as measured by the time course assay. The CD147 inhibitor had an inhibitory effect trend only between 48 to 96 h (*p* > 0.1) and not at shorter or longer time points. The addition of TFPI did not alter this inhibitory pattern (Supplementary Fig. 2B).

### Enhanced migration caused by cancer cell-derived EVs is reversed by dual inhibitor treatment

**Fig. 3:**
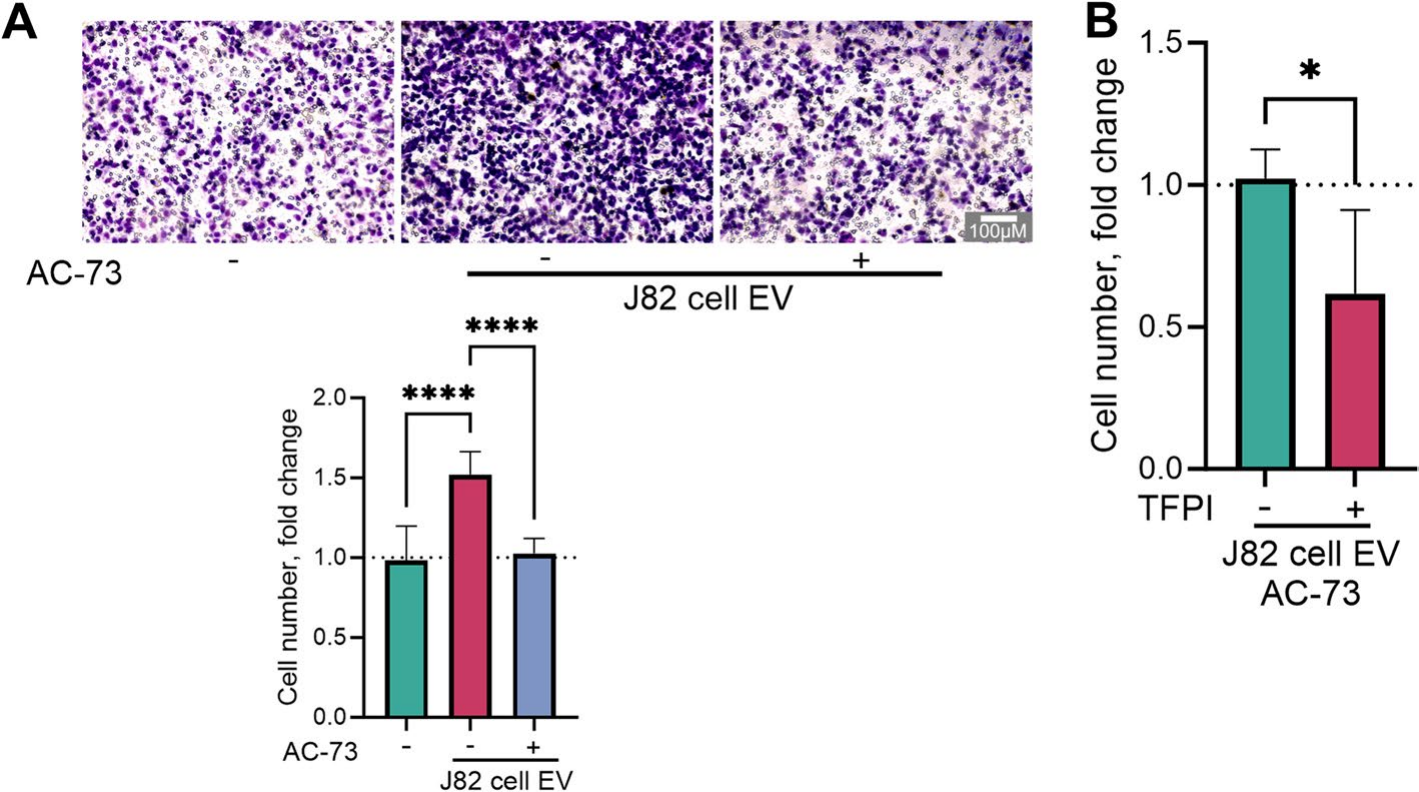
Impact of the combined inhibitors treatment on extracellular vesicle (EV)-induced migration potential. J82 human bladder urothelial carcinoma cells were exposed to 150 µg/ml protein from J82 cell-derived EVs and treated with either 5 µM CD147 inhibitor AC-73 alone (**A**) or a combination of 2.5 µM AC-73 and tissue factor pathway inhibitor (TFPI, 300 ng/ml, **B**) for 16 h. Relative migration was quantified via a Transwell assay utilizing 6–8 images of cells that traversed the microporous membrane per experiment

Treatment with cell-derived EVs increased migration by an average of 32% compared to that of the control, untreated cells (Fig. 3A). The specific inhibitor AC-73 reversed the migration induced by EV back to the level observed in the control untreated cells. Pretreatment of EVs with TFPI along with AC-73 resulted in an additional reduction in migration potential (Fig. 3B).

### TFPI enhances CD147 inhibitor-mediated suppression of MMP activity induced by cancer EVs

**Fig. 4:**
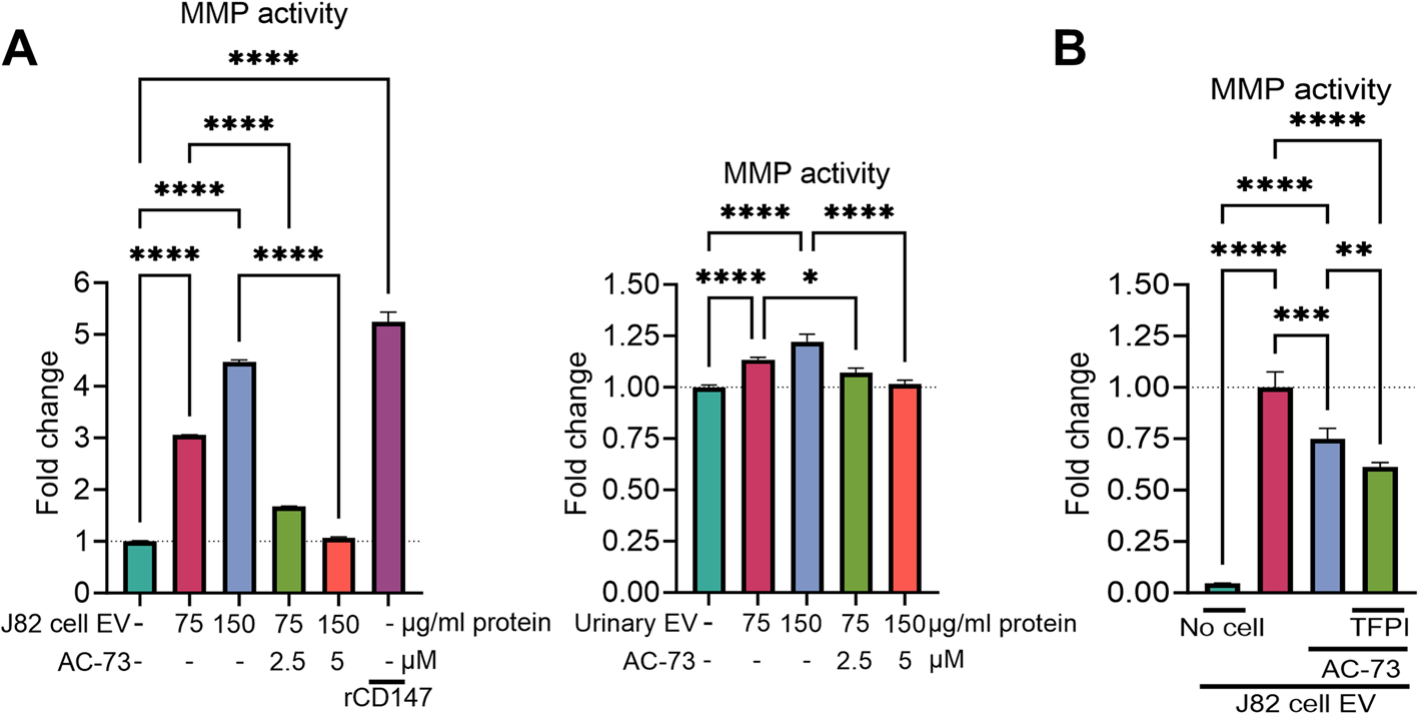
Impact of treatment with the combined inhibitors on extracellular vesicle (EV)-induced matrix metalloproteinases (MMPs) release. J82 human bladder urothelial carcinoma cells were treated with J82 cell-derived EVs or with a pool of isolated EVs from urine samples collected from patients with muscle-invasive bladder cancer (**A**), along with either 2.5 µM or 5 µM of the CD147 inhibitor AC-73, in the absence or presence of tissue factor pathway inhibitor (TFPI, 300 ng/ml, **B**) for 24 h. Recombinant CD147 (rCD147) was used as a positive control to induce MMP activity. The induction of MMPs was measured using colorimetric enzymatic methods

Treatment with EVs derived from J82 cells or collected from urine samples of patients with invasive bladder carcinoma induced dose-dependent MMP activity, which was significantly reduced by treatment with a CD147-specific inhibitor (Fig. 4A). Remarkably, TFPI exerted an additional inhibitory effect on the MMP activity induced by EVs in the presence of AC-73 (-18%, Fig. 4B). We also included a control group, in which the cells were omitted, but the EVs were treated (Fig. 4B). The amount of MMP activity measured in this culture condition was minimal, indicating that the cultured cells, not the EV cargo, were the source of the MMP in the conditioned medium. Our shotgun proteomics analysis of treated cells did not reveal any significant alterations resulting from the influence of cancer EV treatment (Supplementary Fig. 3). This reaffirms that the observed changes stemmed from the modified secretion of MMPs rather than changes in their production.

### Enhanced invasion induced by cancer EVs is reversed by dual inhibitor treatment

**Fig. 5:**
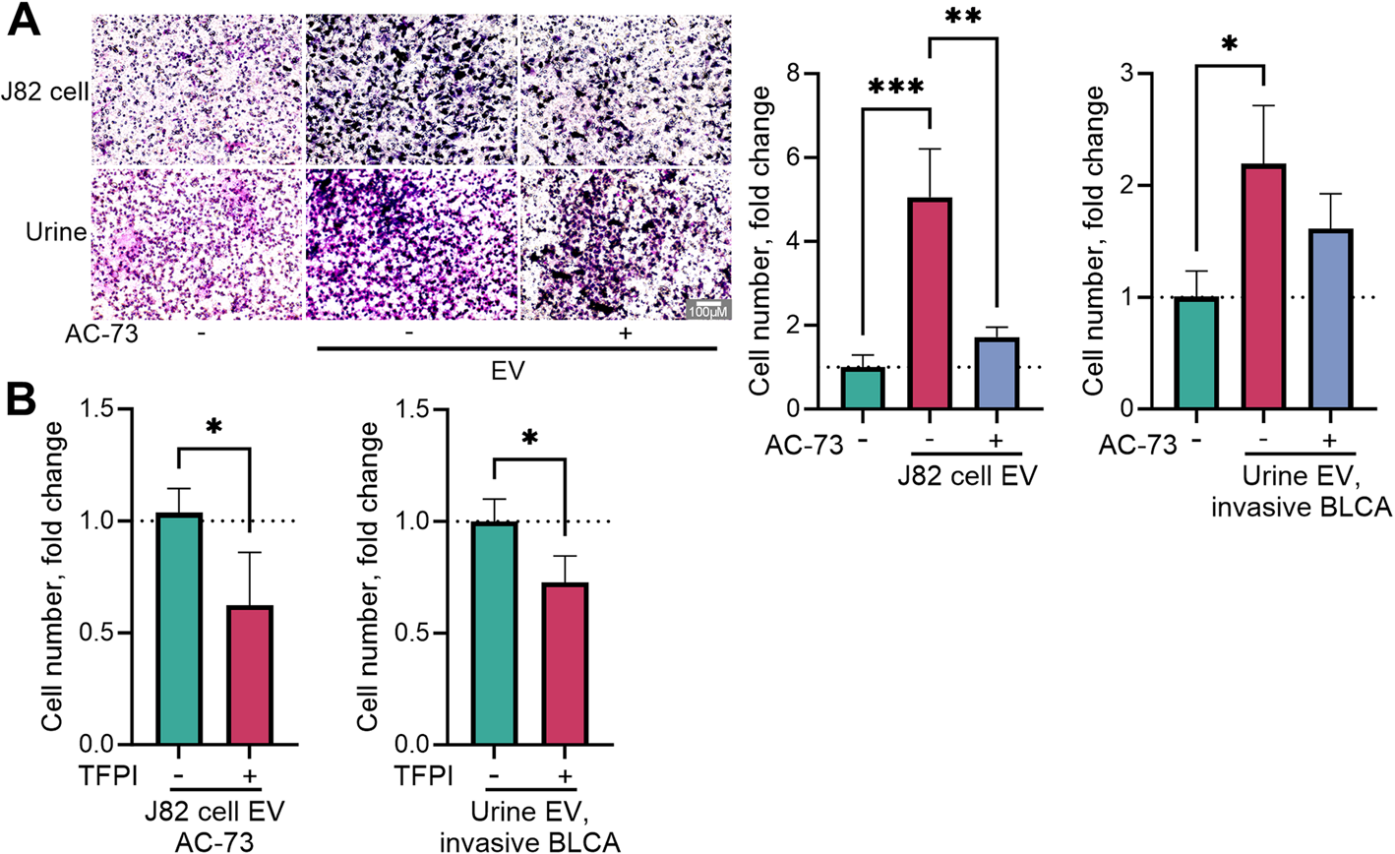
Impact of treatment with the combined inhibitors on extracellular vesicle (EV)-induced invasion potential. J82 human bladder urothelial carcinoma cells were exposed to 150 µg/ml protein from J82 cell-derived EVs or with a pool of isolated EVs from urine samples collected from patients with muscle-invasive bladder cancer, and treated with either 5 µM of the CD147 inhibitor AC-73 alone (**A**) or a combination of 2.5 µM AC-73 and tissue factor pathway inhibitor (TFPI, 300 ng/ml, **B**) for 24 h. Invasion potency was measured via a Transwell assay utilizing 6–8 images of cells that traversed the microporous membrane per experiment

J82 cell supernatant-derived EVs increased cell invasion by 5.6-fold, but the CD147-specific inhibitor effectively countered this effect by reducing cell invasion to only twice the amount observed in the untreated control (Fig. 5A). Adding TFPI to cells treated with AC-73 further reduced cell invasion by 50%, suggesting a potential interaction between TFPI and CD147 (Fig. 5B). This trend was also observed for cancer cell invasion using EVs isolated from urine samples obtained from patients with muscle-invasive BLCA (Fig. 5).

### Dual inhibitor treatment enhances the disruption of EV binding to cells

**Fig. 6:**
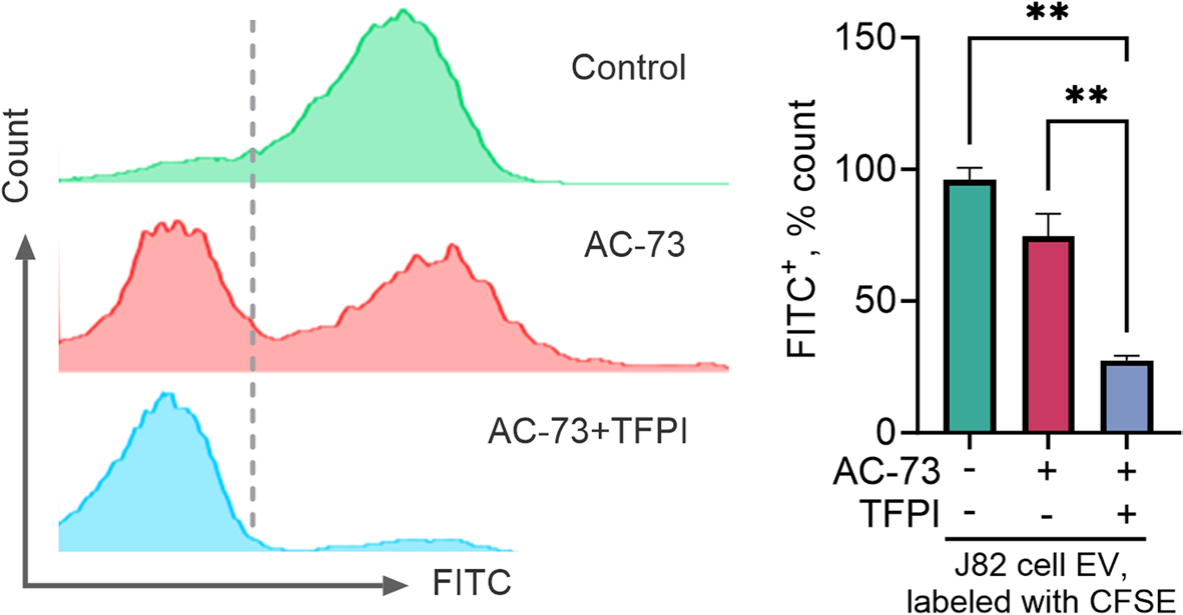
Impact of treatment with the combined inhibitors on the extracellular vesicle (EV) binding capability. J82 human bladder urothelial carcinoma cells were exposed in suspension to carboxyfluorescein succinimidyl ester fluorescent dye (CFSE)-labeled J82 cell-derived EVs and treated with either 5 µM CD147 inhibitor AC-73 alone or a combination of 2.5 µM AC-73 and tissue factor pathway inhibitor (TFPI, 300 ng/ml). The cells were analyzed for green fluorescence in the FITC channel using flow cytometry

A suspended binding assay was conducted to evaluate how dual inhibitor treatment interferes with the interaction of EVs with target cells. In this assay, labeled EVs were allowed to bind briefly to unlabeled suspended J82 cells. The introduction of the CD147 inhibitor AC-73 resulted in a moderate decrease in the fluorescent signal, confirming its ability to interfere with EV-cell surface binding (Fig. 6). However, when TFPI and AC-73 were applied together to the EVs, a more substantial reduction in the fluorescent signal was observed than when AC-73 was used alone. These findings indicate that TFPI enhances the disruptive effect of AC-73 on EV binding to the cell surface (Fig. 6).

## Discussion

In this study, we investigated a novel strategy to disrupt the homotypic interaction of cancer-derived microvesicle-like EVs with cancer cells by simultaneously targeting the highly expressed TF and CD147 molecules. We demonstrated that TFPI, a naturally occurring inhibitor of TF, enhances the suppressive impact of a CD147 inhibitor on cancer cell progression in vitro. Our findings highlight the potential of this interference to significantly inhibit tumor migration, infiltration, and subsequent metastasis.

The detection of the well-established EV markers CD9, CD63, and CD81 in the isolated particles validated the capability of the larger, microvesicle-like EV isolation in this study. The observed differences in size range and marker enrichment between small and large EVs may imply distinct functional roles for these subpopulations. However, this was not the focus of the present investigation. We observed the shedding of tagged CD147 into subpopulations of the examined EVs. These CD147^+^ EVs are likely to represent microvesicles, given that CD147 is predominantly localized to the plasma membrane. Notably, our findings align with a prior study demonstrating CD147 immunoreactivity in microvesicle-like EVs derived from tumor cells, but not in exosomes [34].

We observed significantly higher CD147 expression in invasive versus non-invasive cancer cells, suggesting a potential association between CD147 expression and invasion. Additionally, EVs derived from invasive cells exhibited higher levels of TF activity and CD147 expression compared to those from non-invasive equivalents, highlighting a potential association between these factors in EVs from invasive cancer cells. This phenomenon was also observed in urine samples from patients with invasive BLCA. However, the differences in TF did not reach statistical significance, which is probably due to the limited sample size and the possible test variability due to the different sample matrices.

Urine contains a high concentration of EVs associated with bladder cancer [35, 36]. Previous research on urinary EVs has focused mainly on analyzing their molecular composition in relation to different diseases, while examining their functional potential is still in the early stages. Nevertheless, some studies have provided insights into their pathological function in cancers of the genitourinary system [37–39], which aligns with our findings. Notably, bladder barbotage-derived EVs, which are likely enriched with bladder cancer EVs, exhibited a more pronounced effect compared to urinary-derived EVs [39]. Our study is distinctive in its focus on larger vesicles, which can be more readily separated and are more likely to carry the signature of the parent cells plasma membrane.

We produced model EVs enriched with the TF and CD147 proteins by overexpressing these factors in J82 cells. Testing these vesicles compared to vesicles derived from parental cells demonstrated a significant increase in the degree to which the vesicles induced cellular invasion. The specific interaction mechanism between CD147 and TF remains unknown. However, in the context of infection, high secretion of CD147 by epithelial cells, which is induced by bacterial virulence factors, is associated with upregulation of endothelial TF activation [40]. Studying how these two proteins interact can provide insight into the potential implications of combining seemingly unrelated anti-CD147 and anticoagulation therapies on cancer progression.

Our findings demonstrated that the CD147 inhibitor AC-73 exerts a dose-dependent effect on cell viability, and the addition of TFPI does not influence this effect. The lack of a significant effect of TFPI suggested that its direct influence on cell viability and proliferation may be limited. Prior studies have demonstrated that TFPI inhibits proliferation only at excessive concentrations through an apoptotic mechanism [18, 41] and may indirectly affect tumor growth at lower concentrations by inhibiting angiogenesis through a nonhemostatic mechanism [19, 42]. To elucidate the specific molecular actions of AC-73 and TFPI, we applied concentrations of these compounds that do not interfere with cell viability and proliferation, mirroring higher in vivo relevance.

By employing a specific CD147 inhibitor, our study showed that CD147 plays a significant role as a component in cancer-derived EVs, enhancing their ability to induce migration and invasion. These findings add to the growing body of evidence supporting the significance of CD147 in cancer progression. Previous studies have demonstrated that CD147 is a key cell surface receptor and adhesion molecule for migration and invasion [43]. Monoclonal antibodies or chemical inhibitors specific to CD147 can inhibit the in vitro migration of cancer cells, demonstrating the dependence on CD147 interaction for collective invasion through EVs [43]. Menck et al. [13] showed that tumor-derived microvesicles induce autologous and heterologous human breast cancer invasion through a highly glycosylated form of CD147. Consistent with our observation, this impact was primarily mediated through cellular signal induction rather than enhanced EV uptake. In addition to its involvement in promoting migration and invasion, CD147 in malignant cells also upregulates the release of EVs, as demonstrated by our current study and others [44].

Our study demonstrated that cancer-derived EVs induce autologous MMP expression in J82 cells, contrasting with a previous report indicating that CD147^+^ EVs did not affect MMP expression in breast cancer cell models [13]. These discordant results might be due to the reliance of the previous study on gene expression rather than MMP activity. Additionally, we cannot exclude the possibility of cell-type-specific responses to EVs, which could account for the observed differences. These possibilities are consistent with the observation that CD147 in EVs derived from noncancerous peritoneal cells promotes the invasion of gastric cancer cells [45].

Previous studies have highlighted the essential role of MMP-mediated extracellular degradation and remodeling in angiogenesis [46]. Our findings align with the induction of proangiogenic activities by microvesicles shed by cancer cells through a CD147-mediated mechanism [47]. Evidence also suggests that TF-mediated signaling in cancer cells plays a role in promoting invasion by upregulating the expression of MMPs [48].

The release of plasma membrane-derived vesicles with procoagulant activity was initially demonstrated in cancer cells through in vitro experiments [49]. However, at the clinical level, the link between TF and decreased survival in cancer cannot be solely attributed to the prothrombotic effects of an activated coagulation system. It has been observed that TF and its downstream coagulation proteases are also interconnected with signaling cascades that promote tumor growth, metastasis, and angiogenesis [50–53]. In this study, we showed that EVs originating from invasive urothelial cancer cells are notably enriched with CD147 and TF, aligning with their established recognition as candidate prognostic markers and promising therapeutic targets in cancer. In particular, a meta-analysis comprising several datasets showed the association of CD147 [54], and an immunohistochemical tumor study revealed the association of TF [55] with clinicopathological features of bladder cancer.

Remarkably, both CD147 and the TFPI are under investigation as potential clinical targets [56–58]. Furthermore, we have demonstrated that TFPI released in response to an LMWH can effectively inhibit cancer cell migration induced by EVs derived from malignant effusions [20]. These EVs were characterized as enriched with TF and demonstrated the ability to activate migration-inducing signaling in cancer cells [24]. Therefore, we speculate that TFPI could potentiate the suppressive effect of CD147 inhibitors on the protumoral functions of cancer-derived EVs.

In the present study, TFPI was shown to exert an additional interfering effect when used in combination with a CD147 inhibitor, further suppressing autologous EV-induced cancer cell migration, MMP production, and invasion. The proposed interfering effect of TFPI on the binding and functional capability of CD147 on EVs introduces a novel regulatory mechanism in the context of CD147-mediated processes. By interfering with CD147, TFPI may disrupt key signaling pathways involved in tumor migration and metastasis.

The limited availability of suitable and widely accepted animal models for human bladder cancer poses a challenge for research in this field. This study was conducted with a specific urothelial cancer cell line, and caution is essential when these findings are applied to other types of cancer or in vivo settings. The impact of combining TFPI or its inducers LMWHs with a CD147 inhibitor could be influenced by the specific tumor microenvironment in vivo. Nonetheless, the unique aspects of our study, particularly the integration of urine sample analysis, provide valuable insights within the constraints of currently available models.

## Conclusions

Our findings suggest that combining a CD147 inhibitor with LMWHs, especially those with high TFPI-releasing potency, may be a promising therapeutic approach for urothelial cancer management. This combination can potentially suppress the tumor-promoting actions of cancer-derived microvesicle-like EVs, including migration, MMP secretion, and matrix invasion. The specific molecular mechanisms underlying this enhanced inhibitory effect remain to be fully elucidated, but the combination warrants further investigation in preclinical and clinical studies.

### Supplementary Information

**Additional file 1: Supplementary Table 1.** Demographic characteristics of patients with non-muscle and muscle -invasive bladder carcinoma. **Supplementary Figure 1.** Characterization of extracellular vesicles (EVs) derived from non-invasive RT4 cells. **Supplementary Figure 2.** Impact of treatment with the combined inhibitors in the presence of extracellular vesicles (EVs) on cell viability and proliferation. **Supplementary Figure 3.** Impact of cancer-derived extracellular vesicles (EVs) on cancer cell proteomics.

## Data Availability

All the data supporting the findings of this study are available within the article and in the supplementary information files or from the authors upon request. The mass spectrometry proteomics data have been deposited with the ProteomeXchange Consortium via the PRIDE partner repository with the dataset identifier PXD044680.

## References

[1] Baumann Z, der AufMaur P, Bentires-Alj M (2022). Feed-forward loops between metastatic cancer cells and their microenvironment-the stage of escalation. EMBO Mol Med.

[2] Cocucci E, Racchetti G, Meldolesi J (2009). Shedding microvesicles: artefacts no more. Trends Cell Biol.

[3] Ratajczak MZ, Ratajczak J (2020). Extracellular microvesicles/exosomes: discovery, disbelief, acceptance, and the future?. Leukemia.

[4] Singer SJ (1992). Intercellular communication and cell-cell adhesion. Science.

[5] Raposo G, Stoorvogel W (2013). Extracellular vesicles: exosomes, microvesicles, and friends. J Cell Biol.

[6] Matsumoto Y (2020). Tumor-derived exosomes influence the cell cycle and cell migration of human esophageal cancer cell lines. Cancer Sci.

[7] Tomiyama E (2022). EphA2 on urinary extracellular vesicles as a novel biomarker for bladder cancer diagnosis and its effect on the invasiveness of bladder cancer. Br J Cancer.

[8] Wang X (2021). Exosomes derived from nasopharyngeal carcinoma cells induce IL-6 production from macrophages to promote tumorigenesis. Cell Mol Immunol.

[9] Andrade LNS (2019). Extracellular Vesicles Shedding Promotes Melanoma Growth in Response to Chemotherapy. Sci Rep.

[10] Wortzel I (2019). Exosome-mediated metastasis: communication from a distance. Dev Cell.

[11] Gieseler F (2018). Heterogeneity of microvesicles from cancer cell lines under inflammatory stimulation with TNF-α. Cell Biol Int.

[12] Hisada Y, Sachetto ATA, Mackman N (2022). Circulating tissue factor-positive extracellular vesicles and their association with thrombosis in different diseases. Immunol Rev.

[13] Menck K (2015). Tumor-derived microvesicles mediate human breast cancer invasion through differentially glycosylated EMMPRIN. J Mol Cell Biol.

[14] Tian Y (2018). Protein Profiling and Sizing of Extracellular Vesicles from Colorectal Cancer Patients via Flow Cytometry. ACS Nano.

[15] Yoshioka Y (2014). Ultra-sensitive liquid biopsy of circulating extracellular vesicles using ExoScreen. Nat Commun.

[16] Menck K (2017). Characterisation of tumour-derived microvesicles in cancer patients' blood and correlation with clinical outcome. J Extracell Vesicles.

[17] Spinello I (2019). The small-molecule compound AC-73 targeting CD147 inhibits leukemic cell proliferation, induces autophagy and increases the chemotherapeutic sensitivity of acute myeloid leukemia cells. Haematologica.

[18] Provençal M (2008). Tissue factor pathway inhibitor (TFPI) interferes with endothelial cell migration by inhibition of both the Erk pathway and focal adhesion proteins. Thromb Haemost.

[19] Holroyd EW (2012). Tissue factor pathway inhibitor blocks angiogenesis via its carboxyl terminus. Arterioscler Thromb Vasc Biol.

[20] Gamperl H (2016). Extracellular vesicles from malignant effusions induce tumor cell migration: inhibitory effect of LMWH tinzaparin. Cell Biol Int.

[21] Pham CV (2021). Bovine extracellular vesicles contaminate human extracellular vesicles produced in cell culture conditioned medium when 'exosome-depleted serum' is utilised. Arch Biochem Biophys.

[22] Maïssa N (2017). Strength of Neisseria meningitidis binding to endothelial cells requires highly-ordered CD147/β. Nat Commun.

[23] Muhsin-Sharafaldine MR (2016). Procoagulant and immunogenic properties of melanoma exosomes, microvesicles and apoptotic vesicles. Oncotarget.

[24] Ender F (2020). Tissue factor activity on microvesicles from cancer patients. J Cancer Res Clin Oncol.

[25] Ender F (2019). Detection and Quantification of Extracellular Vesicles via FACS: Membrane Labeling Matters!. Int J Mol Sci.

[26] Steidel C (2021). Biologically active tissue factor-bearing larger Ectosome-like extracellular vesicles in malignant effusions from ovarian cancer patients: correlation with incidence of thrombosis. Int J Mol Sci.

[27] Meier F (2020). diaPASEF: parallel accumulation-serial fragmentation combined with data-independent acquisition. Nat Methods.

[28] Demichev V (2020). DIA-NN: neural networks and interference correction enable deep proteome coverage in high throughput. Nat Methods.

[29] Demichev V (2022). dia-PASEF data analysis using FragPipe and DIA-NN for deep proteomics of low sample amounts. Nat Commun.

[30] Cox J (2014). Accurate proteome-wide label-free quantification by delayed normalization and maximal peptide ratio extraction, termed MaxLFQ. Mol Cell Proteomics.

[31] Zhang X (2018). Proteome-wide identification of ubiquitin interactions using UbIA-MS. Nat Protoc.

[32] Huber W (2002). Variance stabilization applied to microarray data calibration and to the quantification of differential expression. Bioinformatics.

[33] Ritchie ME (2015). limma powers differential expression analyses for RNA-sequencing and microarray studies. Nucleic Acids Res.

[34] Sidhu SS (2004). The microvesicle as a vehicle for EMMPRIN in tumor-stromal interactions. Oncogene.

[35] Linxweiler J, Junker K (2020). Extracellular vesicles in urological malignancies: an update. Nat Rev Urol.

[36] Silvers CR (2017). Characterization of urinary extracellular vesicle proteins in muscle-invasive bladder cancer. Oncotarget.

[37] Yang L (2013). Bladder cancer cell-derived exosomes inhibit tumor cell apoptosis and induce cell proliferation in vitro. Mol Med Rep.

[38] Beckham CJ (2014). Bladder cancer exosomes contain EDIL-3/Del1 and facilitate cancer progression. J Urol.

[39] Franzen CA (2015). Urothelial cells undergo epithelial-to-mesenchymal transition after exposure to muscle invasive bladder cancer exosomes. Oncogenesis.

[40] Schulz A (2018). Protective vascular coagulation in response to bacterial infection of the kidney is regulated by bacterial lipid A and host CD147. Pathog Dis.

[41] Hembrough TA (2004). Identification and characterization of a very low density lipoprotein receptor-binding peptide from tissue factor pathway inhibitor that has antitumor and antiangiogenic activity. Blood.

[42] Hembrough TA (2003). Tissue factor/factor VIIa inhibitors block angiogenesis and tumor growth through a nonhemostatic mechanism. Cancer Res.

[43] Arora K (2005). Extracellular cyclophilins contribute to the regulation of inflammatory responses. J Immunol.

[44] Thakur A (2020). Label-free sensing of exosomal MCT1 and CD147 for tracking metabolic reprogramming and malignant progression in glioma. Sci Adv.

[45] Sugimoto A (2021). EMMPRIN in extracellular vesicles from peritoneal mesothelial cells stimulates the invasion activity of diffuse-type gastric cancer cells. Cancer Lett.

[46] Roy R (2020). Metalloproteinases and their roles in human cancer. Anat Rec (Hoboken).

[47] Millimaggi D (2007). Tumor vesicle-associated CD147 modulates the angiogenic capability of endothelial cells. Neoplasia.

[48] Hahn N (2012). Inducible expression of tissue factor in small-cell lung cancer: impact on morphology and matrix metalloproteinase secretion. J Cancer Res Clin Oncol.

[49] Dvorak HF (1981). Tumor shedding and coagulation. Science.

[50] Fan L (2005). Tissue factor enhances protease-activated receptor-2-mediated factor VIIa cell proliferative properties. J Thromb Haemost.

[51] van den Berg YW (2009). Alternatively spliced tissue factor induces angiogenesis through integrin ligation. Proc Natl Acad Sci U S A.

[52] Ott I (2005). Tissue factor cytoplasmic domain stimulates migration by activation of the GTPase Rac1 and the mitogen-activated protein kinase p38. Circulation.

[53] Gieseler F (2013). Proteinase-activated receptors (PARs) - focus on receptor-receptor-interactions and their physiological and pathophysiological impact. Cell Commun Signal.

[54] Li H, Xu Y (2017). CD147 as a novel biomarker for predicting the prognosis and clinicopathological features of bladder cancer: a meta-analysis. Oncotarget.

[55] Patry G (2008). Tissue factor expression correlates with disease-specific survival in patients with node-negative muscle-invasive bladder cancer. Int J Cancer.

[56] Zhinan, C. A study of CD147-targeted car-t by hepatic artery infusions for very advanced hepatocellular carcinoma. 2019; Available from: https://clinicaltrials.gov/ct2/show/NCT03993743. [Cited 2023 14.08.2023].

[57] Mahlangu JN (2023). A phase 1b/2 clinical study of marstacimab, targeting human tissue factor pathway inhibitor, in haemophilia. Br J Haematol.

[58] Dang TT, Morales JC (2023). Abstract B043: Tissue Factor Pathway Inhibitor (TFPI), a novel invasion and therapy target in glioblastoma multiforme. Cancer Res.

