# Supplementary material for: Targeting cancer-derived extracellular vesicles by combining CD147 inhibition with tissue factor pathway inhibitor for the management of urothelial cancer cells
Source: Cell Commun Signal. 2024 Feb 15;22:129. doi: 10.1186/s12964-024-01508-x (PMC10870545; doi:10.1186/s12964-024-01508-x)
Supplement: Supplementary file 1 — Additional file 1: Supplementary Table 1. Demographic characteristics of patients with non-muscle and muscle -invasive bladder carcinoma. Supplementary Figure 1. Characterization of extracellular vesicles (EVs) derived from non-invasive RT4 cells. Supplementary Figure 2. Impact of treatment with the combined inhibitors in the presence of extracellular vesicles (EVs) on cell viability and proliferation. Supplementary Figure 3. Impact of cancer-derived extracellular vesicles (EVs) on cancer cell proteomics. [file 12964_2024_1508_MOESM1_ESM.docx]

**Supplementary methods**

**LC-MS/MS acquisition**

Mobile phases A and B were water with 0.1% (v/v) formic acid and 0.1% (v/v) acetonitrile, respectively. The liquid chromatography system was coupled to a hybrid TIMS quadrupole TOF mass spectrometer (Bruker timsTOF Flex HT) via a CaptiveSpray nano-electrospray ion source. The mass spectrometer was operated in the diaPASEF mode [56]. The MS settings were set to a scan range of 100-1,700 m/z in positive ion polarity. The TIMS settings were set to an ion mobility range of 0.6-1.6 1/K0, a ramp time of 100 ms, an accumulation time of 100 ms, and a ramp rate of 9.43 Hz. The MS/MS settings were set to a mass range of 400 to 1,201 Da and a mobility range of 0.6-1.6 1/K0. We use a fixed dia-PASEF window width of 26.0 Da, with 1.0 Da mass overlap and 0.0 1/K0 mobility overlap. The method was set to 32 mass steps and 1.0 mobility window per cycle. The entire method had an estimated cycle time of 1.80 seconds.

The acquired diaPASEF mass spectra were processed using the DIA-NN v1.8.1 software tool [57, 58]. The software was used in the high precision LC mode, with RT-dependent cross normalization enabled, smart profiling for Library generation, and single-pass mode as neural network classifier. Mass accuracy and MS1 accuracy were set to 10.0, and the scan window was set to 0 (DIA-NN automatically determines scan window width). The ‘Match between runs’ option was enabled. The human UniprotKB/swiss-prot database (downloaded on 12/22/2021) was used for deep learning based *in silico* spectral library generation. The protease setting was set to trypsin/P, while 1.0 missed cleavage was accepted. The maximum number of variable modifications was set to 0.0, with N-terminal methionine excision and cysteine carbamidomethylation were enabled as fixed modifications. The peptide length range was set to 7-30, precursor charge range to 2-4, precursor m/z range to 100-1,700, and fragment ion m/z range to 400-1,201.

**S****upplementary Table 1.** Demographic characteristics of patients with non-muscle and muscle -invasive bladder carcinoma

|  | Non-muscle invasive  n=17 | Muscle invasive  n=10 | *p* |
| --- | --- | --- | --- |
| Age, y | 71.53 + 11.28 | 73.30 + 7.22 | 0.66 |
| Gender, female/male | 2 / 15 | 4 / 6 | 0.09 |
| Creatinine, μmol/L | 116.41 + 66.49 | 102.24 + 40.31 | 0.55 |
| GFR, ml/min | 65.24 + 25.77 | 65.40 + 20.34 | 0.99 |
| Urine albumin, g/L | 41.28 + 38.39 | 78.55 + 29.77 | 0.11 |


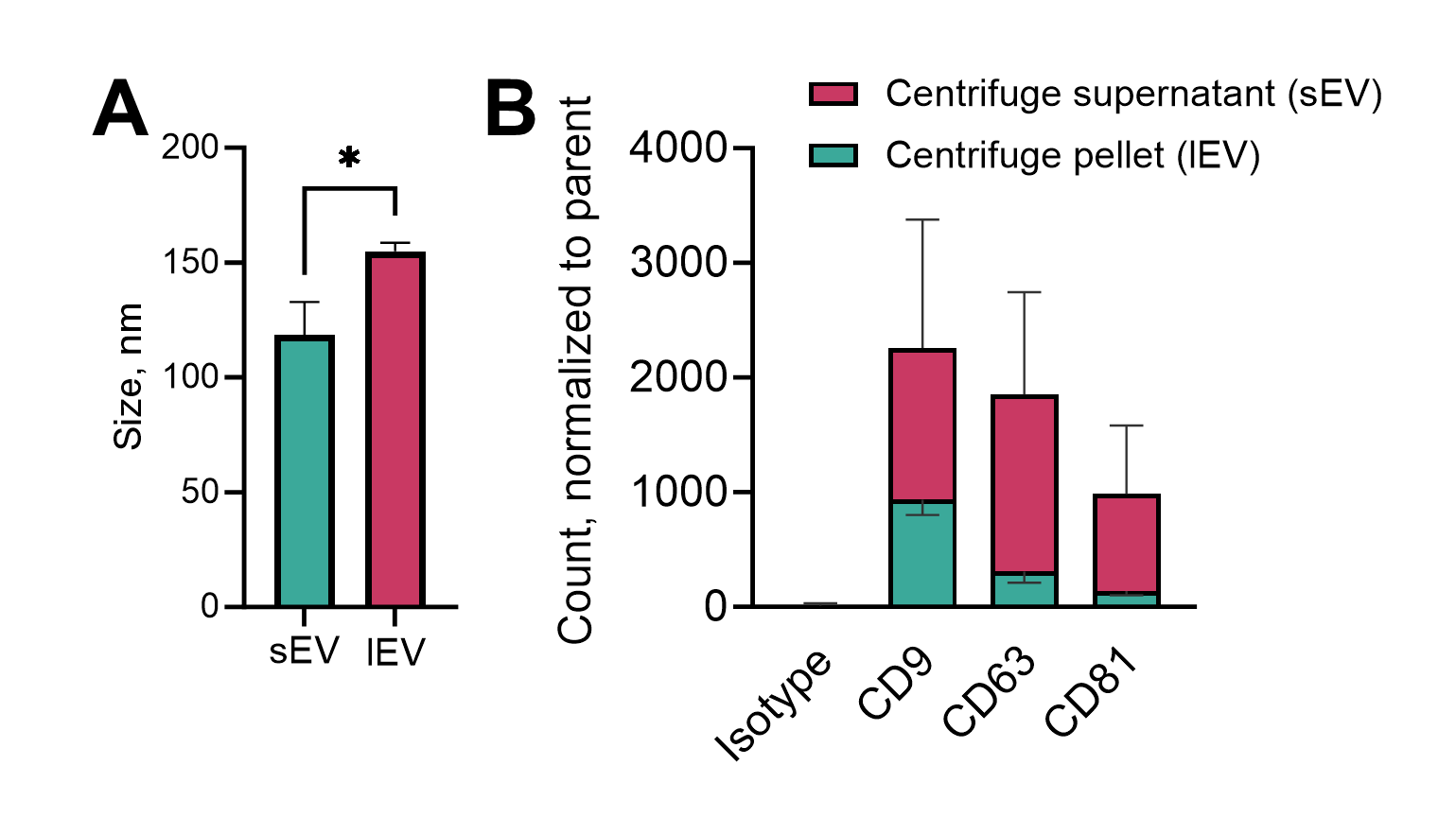


**Supplementary Figure 1. Characterization of extracellular vesicles (EVs) derived from non-invasive RT4 cells**. The size distribution of EVs isolated from the supernatant (sEVs) and pellet (lEVs) was determined using nanoparticle tracking analysis (NTA) (A). The presence of tetraspanin EV markers (CD9, CD63, and CD81) on sEVs and lEVs was assessed by flow cytometry (B).


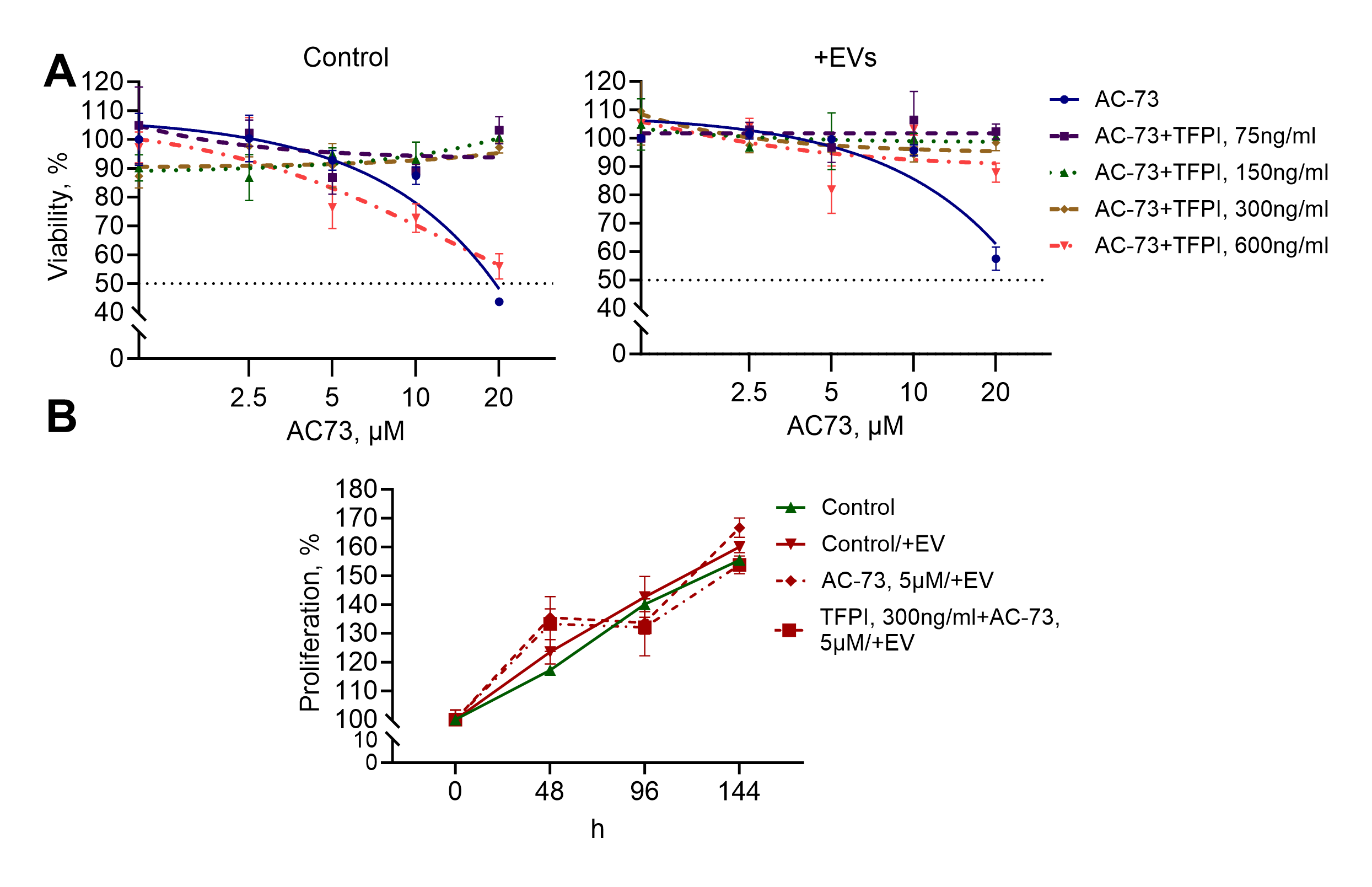


**Supplementary Figure 2. Impact of treatment with the combined inhibitors in the presence of extracellular vesicles (EVs) on cell viability and proliferation.** J82 human bladder urothelial carcinoma cells were exposed to 150 µg/ml protein from J82 cell-derived EVs and treated with 0–20 µM CD147 inhibitor AC-73 alone or a combination of AC-73 and 75-600 ng/ml tissue factor pathway inhibitor (TFPI) for 24 h (**A**). Cell viability was determined using the MTS tetrazolium reduction assay with 10% tetrazolium solution incubated for 3 h. The percentage of proliferation in the presence of AC-73 and TFPI at indicated time points (**B**).


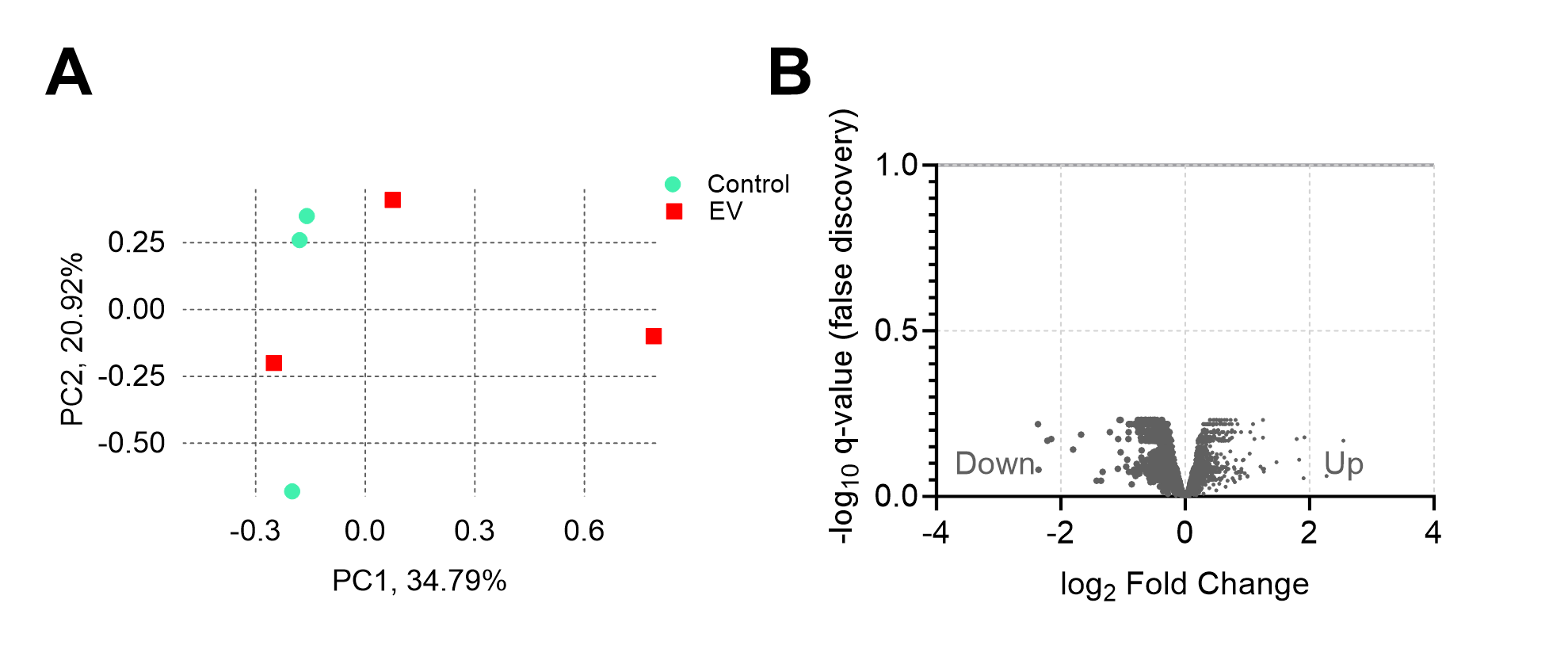


**Supplementary Figure 3. Impact of cancer-derived extracellular vesicles (EVs) on cancer cell proteomics.** Principal component analysis (**A**) and volcano plot (**B**) of identified proteins between untreated and EV-treated cells. Human bladder urothelial carcinoma cells J82 were exposed to 150 µg/ml protein from J82 cell-derived EVs for 48 h. A total of 5,982 proteins were identified. Comparison between samples yielded no significantly differentially expressed proteins. Data are available via ProteomeXchange with the identifier PXD044680.

**The proteomics data**

The mass spectrometry data can be accessed at <https://www.ebi.ac.uk/pride/login> with the following reviewer account:

Username: reviewer_pxd044680@ebi.ac.uk

Password: WeFiROhy
